# Incidental pulmonary nodules in oral squamous cell carcinoma – A retrospective cohort study

**DOI:** 10.1007/s10006-026-01601-0

**Published:** 2026-07-13

**Authors:** Matthew Gilmore, Aleeza Baker, Omar Breik

**Affiliations:** 1 Royal Brisbane and Women’s Hospital & Griffith University, Brisbane, Australia; 2 Royal Brisbane and Women’s Hospital, Brisbane, Australia; 3 Royal Brisbane and Women’s Hospital & University of Queensland, Brisbane, Australia

**Keywords:** Pulmonary nodules, Lung metastases, Computed tomography chest, Oral cancer, Head and neck cancer

## Abstract

**Purpose:**

Incidental pulmonary nodules are frequently identified on staging imaging for oral cavity squamous cell carcinoma (SCC). These may represent metastases, lung primaries, or benign lesions. Existing risk stratification guidelines are not specific to head and neck cancer. This retrospective cohort study evaluated survival differences between patients with and without pulmonary nodules and explored follow-up imaging practices.

**Methods:**

This single-centre retrospective cohort study included adults with oral mucosal SCC managed with curative intent between 2011 and 2018. Clinical, demographic, and radiographic data, including nodule characteristics and follow-up imaging, were extracted from electronic records. Primary outcomes were overall survival and metastatic disease–related death, analysed using Fisher’s exact test, Kaplan–Meier survival with log-rank testing, and multivariable Cox regression.

**Results:**

Of 4563 clinic patients screened, 372 met inclusion criteria (mean age 58.3 years, 62.9% male). Pulmonary imaging was available in 84.1% of patients; among those with baseline thoracic imaging, pulmonary nodules were present in 25.6%, predominantly measuring < 6 mm and radiographically indeterminate. Pulmonary nodules were not independently associated with metastatic disease-related death or reduced overall survival within this cohort. Nodule size and multiplicity did not demonstrate statistically significant associations with metastatic disease-related death. Older age, nodal disease, and perineural invasion were independently associated with poorer survival.

**Conclusion:**

Pulmonary nodules were common but were not independently associated with overall survival or metastatic mortality within this cohort. Established oncologic factors remained the dominant prognostic determinants. Small incidental pulmonary nodules identified on staging imaging should not, in isolation, alter curative treatment intent. Further prospective studies are required to clarify the prognostic significance of pulmonary nodules, particularly larger or radiologically suspicious lesions, and to determine the role of surveillance imaging strategies.

## Introduction

The lungs are a common site for distant metastatic disease in head and neck cancer [1]. The presence of pulmonary metastases or synchronous pulmonary tumours can change clinical management, and thus requires adequate and thorough clinical screening [2–4]. Chest computed tomography (CT) has surpassed the posterior-anterior chest radiograph in the staging algorithm for head and neck cancer [4]. The technological increase in the spatial resolution of chest CT has led to the increased detection of smaller incidental lesions [5–7].

Current data estimates that 14% to 30% of head and neck cancer patients have pulmonary nodules diagnosed at initial imaging [8, 9]. Pulmonary nodules are focal lung parenchymal lesions found on imaging and are surrounded by aerated lung; they are traditionally 3 cm or less in diameter [7]. Pulmonary nodules may be due to benign causes such as granulomatous or inflammatory diseases, or healed infections, all unrelated to malignancy [6].

There is currently no clear algorithm regarding the management of pulmonary nodules specific to the head and neck cancer cohort [9]. Most centres use generalised guidelines (such as the British Thoracic Society Guideline [5, 10]) that are not specific to the head and neck population. Despite shared risk factors between head and neck cancer and primary lung cancer, many pulmonary nodules are benign [11]. Increased CT utilisation has led to more incidental pulmonary nodules, and distinguishing benign from malignant disease remains clinically challenging with important management implications [9].

Management of incidental pulmonary nodules has been addressed in the broader respiratory and thoracic imaging literature [5, 7]. Contemporary regional clinical guidelines emphasise structured risk stratification of pulmonary nodules based on radiographic characteristics, patient risk factors, and interval change [12]. This approach recognises that most incidentally detected nodules, particularly small lesions, are benign and should not automatically be assumed to represent malignancy [7].

Uncertainty remains regarding the true prognostic significance of incidental pulmonary nodules in patients with oral cavity squamous cell carcinoma. It is unclear whether the presence of nodules at staging imaging is associated with increased risk of metastatic disease or decreased survival, and whether specific radiographic features of nodules can reliably identify patients at higher risk of adverse outcomes [13].

This retrospective cohort study aims to determine the incidence and clinical significance of incidental pulmonary nodules detected on staging imaging in patients with oral cavity squamous cell carcinoma. The primary objective is to evaluate whether the presence of pulmonary nodules was associated with death from metastases or reduced overall survival. Secondary objectives include describing the radiographic characteristics of pulmonary nodules and assessing whether follow-up imaging practices influence patient outcomes.

## Methods

### Patient selection

This single-centre retrospective cohort study was conducted at a tertiary institution in Brisbane, Australia. Patients were identified from the Royal Brisbane and Women’s Hospital (RBWH) Head and Neck Multidisciplinary Team (MDT) clinic appointment scheduling records. All patients presented as a “new case” or “new case discussion” at the RBWH Head and Neck MDT during the study period were considered for inclusion. Eligible patients included those with a documented squamous cell carcinoma of the oral mucosa, managed through the RBWH Head and Neck MDT and treated with curative intent. Treatment modality was categorised into five groups: surgery alone, surgery followed by adjuvant radiotherapy, surgery followed by adjuvant chemoradiotherapy, primary radiotherapy/chemoradiotherapy, and other treatment modalities. Treatment decisions were made through multidisciplinary team discussion and delivered with curative intent. The study included patients presenting between 1 July 2011 and 31 December 2018, allowing a full 60 months of follow-up to be observed.

Patients were excluded if they were: i. younger than 18 years; ii. treated with palliative intent; or, iii. pregnant at the time of diagnosis or initial MDT discussion.

### Data collection

Clinical and radiographic data were extracted from electronic medical records, radiology reports, and multidisciplinary team documentation. Demographic variables collected included age at diagnosis, gender, smoking status, alcohol use, Aboriginal or Torres Strait Islander status, and relevant comorbidities. Tumour-related variables included primary tumour site, T stage, N stage, pathological adverse features and treatment modalities.

Radiographic reports from staging imaging were reviewed to determine the presence or absence of pulmonary nodules. Radiological characteristics were extracted from contemporaneous radiology reports where documented. No dedicated radiological re-review was performed as part of this study, and imaging descriptors were limited to those recorded in routine clinical practice. Patients without available baseline thoracic imaging were excluded from analyses comparing patients with and without pulmonary nodules, as pulmonary nodule status could not be determined in these individuals. Consequently, comparative survival and prognostic analyses relating to pulmonary nodules were restricted to patients with available staging chest imaging. When nodules were identified, additional data were recorded regarding nodule size, number, and anatomical location within the lungs. Nodule size was categorised by maximal diameter and grouped into clinically relevant categories (< 6 mm, 6–8 mm, and > 8 mm), consistent with commonly used pulmonary nodule risk stratification frameworks.

Follow-up imaging data were also recorded where available. Repeat chest CT imaging was considered ‘appropriate follow-up’ if performed within six months of the initial detection of a pulmonary nodule, or as suggested by contemporaneous clinical documentation. Imaging performed beyond this interval or in the absence of documented clinical intent to monitor the nodule was classified as ‘non-appropriate follow-up’.

Clinical records of all 4563 new patients of the RBWH Head and Neck MDT during the study inclusion period were reviewed. 15 patients were excluded due to expired or unavailable medical records, 44 were excluded as they had definitive metastatic disease, 213 were excluded due to some or all of their treatment occurring at another facility, and the majority were excluded due to having either a non-squamous cell carcinoma diagnosis, or having cancer outside the oral cavity. This yielded 372 patients treated for oral squamous cell carcinoma.

### Outcome measures

The primary outcome of interest were overall survival and death attributable to metastatic disease. Overall survival was defined as the time from primary treatment to death or last clinical review. Survival time was calculated in 28-day months to maintain consistency across the dataset. Cause of death was determined through review of available clinical records, multidisciplinary team documentation, imaging reports, and death-related correspondence contained within the electronic medical record. Deaths were retrospectively classified by the primary investigator as attributable to metastatic disease, attributable to locoregional recurrence, or due to other causes. Death attributable to metastatic disease was assigned when available documentation indicated distant metastatic progression as the principal cause of death, based on radiological, histopathological, or treating clinician assessment where available. Deaths resulting from uncontrolled locoregional disease in the absence of documented distant metastases were classified separately as deaths due to locoregional recurrence. Classification was performed retrospectively by the principal investigator based on available documentation.

Secondary outcomes included radiographic characteristics of pulmonary nodules and the relationship between follow-up imaging and clinical outcomes.

### Statistical analysis

Associations between pulmonary nodules and death due to metastatic disease were examined using contingency tables. Due to the relatively small sample sizes in some subgroup analyses, Fisher’s exact test was used to assess statistical significance.

Overall survival was analysed using Kaplan–Meier survival analysis, and differences between groups were assessed using the log-rank test. Multivariable Cox proportional hazards modelling was performed to identify independent predictors of overall survival. Variables were selected a priori based on established clinical relevance and prior literature regarding prognostic factors in oral cavity squamous cell carcinoma. The final model incorporated age at diagnosis, pulmonary nodule status, T stage group, N stage group, perineural invasion, lymphovascular invasion, and margin status. Treatment modality and extranodal extension were considered but not included in the final model due to concerns regarding collinearity with tumour stage and incomplete data availability, respectively. Depth of invasion was not consistently available within the historical cohort and could not be reliably analysed. Statistical significance was defined as a p-value of less than 0.05.

Data was collected into a de-identified Microsoft Excel (version 2602) RRID: SCR_016137 database; statistical analyses were performed using IBM SPSS Statistics Version 29.0.0.0 (241) RRID: SCR_016479. Kaplan–Meier survival figures were generated using R Project for Statistical Computing RRID: SCR_001905 (version 4.6.0) and the Survminer package (RRID: SCR_021094).

This study was conducted in accordance with the guidelines as outlined in The Strengthening the Reporting of Observational Studies in Epidemiology (STROBE) Statement [14]. Clinical trial number: not applicable.

## Results

Of the 372 patients included for analysis, the cohort had a mean age of 58.3 years and was predominantly male (62.9%). Treatment modality consisted of surgery alone in 212 patients (57.0%), surgery followed by radiotherapy in 104 (28.0%), surgery followed by chemoradiotherapy in 30 (8.1%), primary radiotherapy or chemoradiotherapy in 15 (4.0%), and other treatment approaches in 11 patients (3.0%). Baseline characteristics of the study population are summarised in Table 1.

**Table 1:** Pulmonary nodules stratified by patient demographics, disease characteristics

Characteristic	No Nodules (*n* = 233)	Nodules Present (*n* = 80)	*p*-value
Age, mean ± SD	58.5 ± 14.2	61.6 ± 14.2	*p* = 0.090
Male gender, n (%)	143 (61.4%)	49 (61.3%)	*p* = 0.984
Smoking status			
Never	39 (28.1%)	11 (22.4%)	*p* = 0.516
Former	42 (30.2%)	19 (38.8%)	
Current	58 (41.7%)	19 (38.8%)	
Any History of Smoking	100 (71.9%)	38 (77.6%)	*p* = 0.445
Alcohol Use			
None	40 (29.4%)	13 (27.7%)	*p* = 0.892
Light	38 (27.9%)	12 (25.5%)	
Moderate	18 (13.2%)	8 (17.0%)	
Heavy	35 (27.2%)	14 (29.8%)	
Primary tumour site			
Tongue	109 (46.8%)	42 (52.5%)	*p* = 0.139
Floor of mouth	38 (16.3%)	17 (21.3%)	
Buccal mucosa	20 (8.6%)	4 (5.0%)	
Mandibular alveolus	22 (9.4%)	7 (8.8%)	
Maxillary alveolus	23 (9.9%)	2 (2.5%)	
Retromolar trigone	11 (4.7%)	8 (10.0%)	
Palate	3 (1.3%)	0 (0%)	
Intraosseous mandible	3 (1.3%)	0 (0%)	
T stage group			
T1–T2	130 (56.8%)	43 (53.8%)	*p* = 0.640
T3–T4	99 (43.2%)	37 (46.3%)	
N stage group			
N0	182 (78.4%)	57 (71.3%)	*p* = 0.190
N+	50 (21.6%)	23 (28.8%)	
Treatment group			
Surgery Alone	128 (54.9%)	43 (53.8%)	*p* = 0.941
Surgery + Radiotherapy	69 (29.6%)	24 (30.0%)	
Surgery + Chemoradiotherapy	20 (8.6%)	6 (7.5%)	
Primary Radiotherapy or Chemoradiotherapy	9 (3.9%)	3 (3.8%)	
Other	7 (3.0%)	4 (5.0%)	
Follow-up duration (months), median	Median follow-up duration calculated using reverse Kaplan–Meier: 60 months (95% CI 59.99–60.01)		

Statistical tests used include t-test / Mann-Whitney for continuous variables and Chi-square or Fisher for categorical variables.Percentages are column percentages based available data for each variable: Smoking data available for 188 patients, Alcohol data available for 183 patients. Histopathological variables (PNI, LVI, margin status and ENE) were incompletely available and are reported separately

Pulmonary imaging was available in 84.1% of patients. Patients without available baseline thoracic imaging were not included in comparative analyses examining the prognostic significance of pulmonary nodules. Imaging was more frequently performed in older patients (mean age 59.3 vs. 53.3 years, *p* = 0.017) and those with advanced primary tumours (T3/T4) (χ² = 9.45, *p* = 0.002) but was not independently associated with sex or nodal stage.

Histopathological data were available for a subset of patients (Table 2). Perineural invasion was identified in 102/269 (37.9%) patients and lymphovascular invasion in 19/264 (7.2%). Neither PNI (41.2% vs. 36.8%, Fisher’s exact *p* = 0.564) nor LVI (3.0% vs. 8.6%, Fisher’s exact *p* = 0.172) were associated with pulmonary nodule status. Margin status demonstrated a non-significant trend toward differing distributions between groups (χ² *p* = 0.059).

**Table 2:** Histopathological characteristics according to pulmonary nodule status

Characteristic	No Nodules (*n* = 233)	Nodules Present (*n* = 80)	*p*-value
Perineural invasion			
Present	74 (36.8%)	28 (41.2%)	*p* = 0.564
Absent	127 (63.2%)	40 (58.8%)	
Lymphovascular invasion			
Present	17 (8.6%)	2 (3.0%)	*p* = 0.172
Absent	181 (91.4%)	65 (97.0%)	
Margin status			
Clear (≥ 5 mm)	110 (53.1%)	34 (46.6%)	*p* = 0.059
Close (1 mm: <5 mm)	78 (37.7%)	37 (50.7%)	
Involved (≤ 1 mm)	19 (9.2%)	2 (2.7%)	

Percentages are column percentages based on available data for each variable. Perineural invasion data were available for 269 patients, lymphovascular invasion data were available for 264 patients, and margin status was available for 280 patients. Statistical tests included Fisher’s exact test for binary variables and Chi-square testing for margin status

Pulmonary nodules were found in 80 patients on baseline imaging, corresponding to an incidence of 25.6%. The majority of nodules identified were either small and radiographically indeterminate or lacked clinical characterisation. Radiographic characteristics of identified pulmonary nodules are presented in Table 3.

Among patients with available radiographic measurements, most nodules measured less than 6 mm in maximal diameter. Specifically, 82.6% of nodules were smaller than 6 mm, 13.0% were between 6 and 8 mm, and 4.3% were greater than 8 mm. In terms of number, nodules were present as a single lesion in 57.3% of patients and as multiple nodules in 42.7%. The anatomical distribution of nodules demonstrated a predominance in the upper lobes versus the lower lobes (49.2% and 43.4%, respectively). Nodules arising within the middle lobe were uncommon and accounted for 7.2% of cases.

**Table 3:** Radiographic characteristics of pulmonary nodules

Nodule characteristic	*n* (%)
Total patients with nodules	80
Number of nodules	
Single	43 (57.3%)
Multiple	32 (42.7%)
Nodule size	
<6 mm	57 (82.6%)
6–8 mm	9 (13.0%)
>8 mm	3 (4.3%)
Lobar location	
Right Upper Lobe	21 (30.4%)
Right Middle lobe	5 (7.2%)
Right Lower Lobe	21 (30.4%)
Left Upper Lobe	13 (18.8%)
Left Lower Lobe	9 (13.0%)
Radiographic appearance	
Solid	-
Ground-glass	1 (5.9%)
Calcified	16 (94.1%)
Follow-up CT performed	
Performed	47 (58.8%)
Not performed	33 (41.3%)

Percentages were calculated using available data for each variable

During the follow-up period, 107 patients had a recurrence of cancer, of which 25 patients (6.7%) had pulmonary recurrence as the primary site of recurrence; 72 deaths occurred, representing 19.4% of the study population. Cause of death data were available for a subset of patients and were categorised as ‘death due to metastatic disease’ or ‘death from other causes’. The median follow-up duration for surviving patients was approximately 48 months.

Among patients with pulmonary nodules who subsequently died (and for whom cause of death information was available) (*n* = 33), death from metastatic disease occurred in 10 patients (30.3%). In comparison, 23 patients (69.7%) died from other causes. The presence of pulmonary nodules was not independently associated with an increased likelihood of death from metastatic disease.

When radiographic characteristics of nodules were examined, no statistically significant differences in metastatic death were observed according to nodule size or multiplicity. When nodules were stratified by size, death from metastases occurred in 5 of 16 patients (31.3%) of those with nodules measuring 8 mm or less, whereas no metastatic deaths were observed among the 3 patients with nodules greater than 8 mm. This difference did not reach statistical significance using Fisher’s exact testing (*p* = 0.53).

Similarly, the number of nodules was not independently associated with death from pulmonary metastases (Table 4). Among patients with single nodules, metastatic disease accounted for death in two of ten patients (20.0%), compared to three of nine patients (33.3%) with multiple nodules (Fisher’s exact test *p* = 0.63).

**Table 4:** Nodule characteristics according to death from metastatic disease status

Variable	Metastatic Death *n* (%)	Other Cause *n* (%)	*p*-value
Nodule size ≤8 mm	5 (31.3%)	11 (68.8%)	0.53
Nodule size > 8 mm	0 (0.0%)	3 (100.0%)	
Single nodule	2 (20.0%)	8 (80.0%)	0.63
Multiple nodules	3 (33.3%)	6 (66.7%)	
Follow-up CT performed	7 (33.3%)	14 (66.7%)	0.71
No follow-up CT	3 (25.0%)	9 (75.0%)	
Appropriate timing of follow-up imaging	0 (0.0%)	4 (100%)	0.26
Inappropriate timing of follow-up Imaging	7 (41.2%)	10 (58.8%)	

Percentages were calculated using available data for each variable

**Fig. 1:**
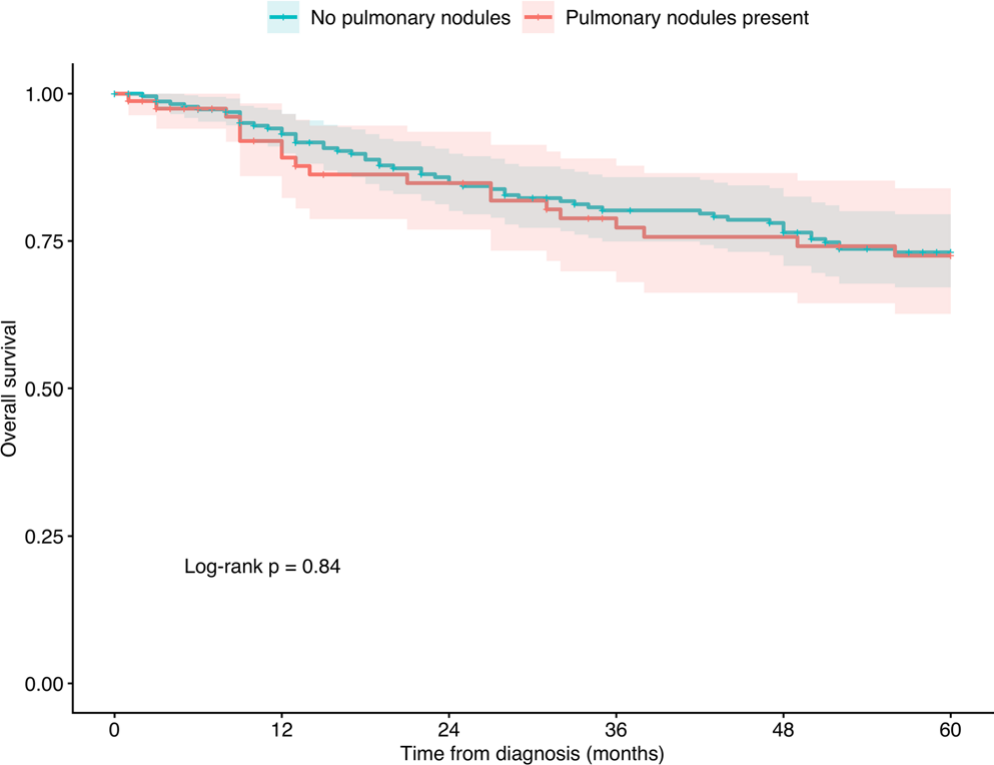
Kaplan-Meier overall survival – pulmonary nodules vs no nodules

**Fig. 2:**
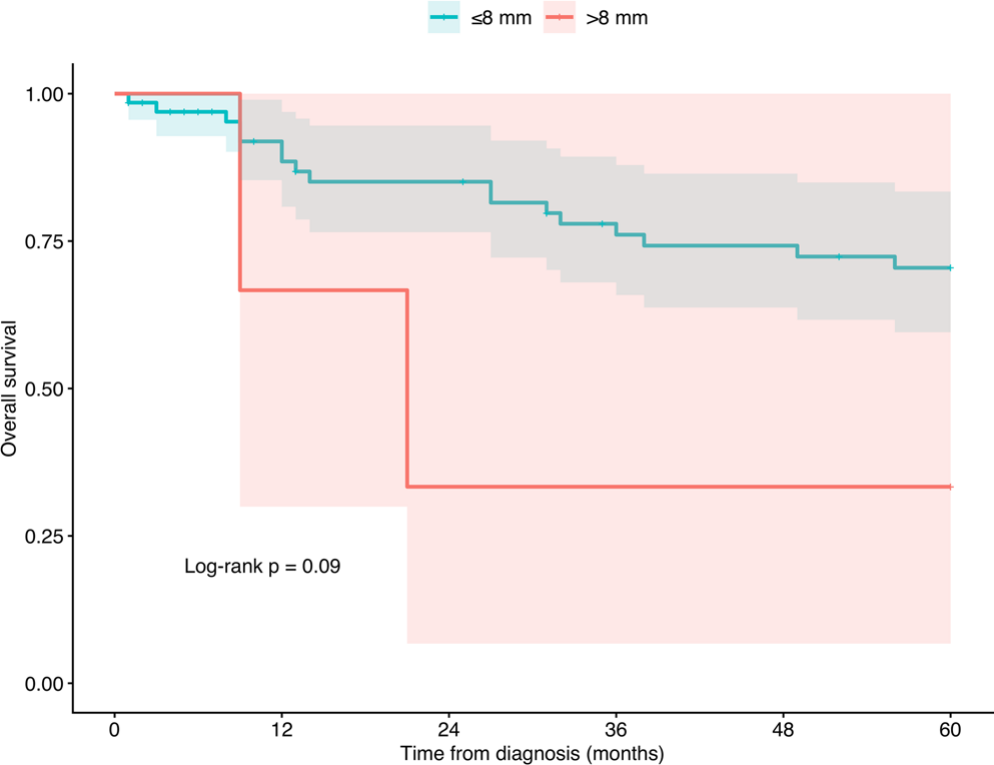
Kaplan–Meier overall survival stratified by pulmonary nodule size (≤ 8 mm vs > 8 mm). Analysis was restricted to patients with available nodule size measurements (*N* = 69)

Of all participants, mean overall survival was 49.5 months for those with pulmonary nodules, and 50.7 months for those without nodules, as shown in the Kaplan-Meier survival analysis in Fig. 1. When survival was stratified by size for those with nodules, mean overall survival was 49.4 months for patients with nodules measuring 8 mm or less and 37.0 months for patients with nodules greater than 8 mm (Fig. 2). Although patients with nodules greater than 8 mm demonstrated numerically poorer overall survival, this difference did not reach statistical significance on log-rank testing (*p* = 0.09). This analysis was limited by the very small number of patients with nodules greater than 8 mm (*n* = 3).

**Table 5:** Multivariable cox proportional hazards model for overall survival

Variable	Hazard Ratio	95% CI	*p*-value
Pulmonary nodules	0.91	0.51–1.64	0.753
Age (per year)	1.06	1.04–1.08	< 0.001
T stage (T3–T4 vs. T1–T2)	1.77	0.98–3.21	0.060
N stage (N + vs. N0)	2.04	1.15–3.62	0.014
PNI	2.50	1.42–4.43	0.002
LVI	1.71	0.79–3.72	0.176
Margin status	1.23	0.85–1.80	0.274

Expanded multivariable Cox regression analyses incorporating additional pathological risk factors were performed to evaluate whether pulmonary nodules remained prognostically significant after adjustment for established clinicopathological variables (Table 5). After adjustment for age, tumour stage, nodal stage, perineural invasion, lymphovascular invasion and margin status, the presence of pulmonary nodules remained non-significant (HR 0.91, 95% CI 0.51–1.64, *p* = 0.753). Perineural invasion emerged as an independent predictor of poorer overall survival (HR 2.50, 95% CI 1.42–4.43, *p* = 0.002), while nodal disease also remained independently associated with mortality (HR 2.04, 95% CI 1.15–3.62, *p* = 0.014). In contrast, lymphovascular invasion and margin status were not independently associated with overall survival.

CT chest imaging after initial staging was performed in 47 patients, representing 58.8% of patients with pulmonary nodules. Among patients with pulmonary nodules who subsequently died and had a documented cause of death (*n* = 33), follow-up imaging was performed in 21 patients (63.6%); 12 patients (36.4%) did not undergo follow-up imaging. Death from metastases occurred in seven of the 21 patients (33.3%) who underwent follow-up imaging and in three of the 12 patients (25.0%) who did not undergo follow-up imaging. This difference was not statistically significant (Fisher’s exact test *p* = 0.71).

Only a small number of patients met criteria for their follow-up imaging to be deemed to be within appropriate timeframes. Within this subgroup, death from metastatic disease occurred in seven of seventeen patients (41.2%) who did not undergo appropriate follow-up imaging. In contrast, no deaths from metastases occurred among the four patients who underwent appropriate follow-up imaging within 6 months. Only four patients underwent follow-up imaging within the predefined appropriate timeframe. Although no metastatic deaths were observed in this small subgroup, the number of patients was insufficient to permit meaningful statistical inference. Consequently, no robust conclusions can be drawn regarding the relationship between follow-up imaging practices and oncologic outcomes in this cohort.

## Discussion

Pulmonary nodules were common and were identified in approximately one-quarter of oral cavity SCC patients undergoing staging imaging in this study. Importantly, most lesions were small incidental findings, with over 80% measuring less than 6 mm in maximal diameter and only three lesions exceeding 8 mm. Consequently, the findings of this study primarily relate to incidental small pulmonary nodules rather than clinically suspicious pulmonary lesions.

Baseline thoracic imaging was not available for all patients. Imaging was more frequently performed in older patients and those with more advanced primary tumours, introducing the potential for ascertainment bias. Although comparative analyses were restricted to patients with available chest imaging, the possibility remains that the imaged cohort differed systematically from patients who did not undergo thoracic imaging.

This study found that pulmonary nodules at baseline imaging were not independently associated with reduced overall survival or an increased risk of death from distant metastatic disease. Furthermore, within the subgroup of patients with nodules, radiographic features such as nodule size and multiplicity were not significantly associated with death from metastatic disease (*p* = 0.53, and *p* = 0.63 respectively). These findings suggest most nodules may represent incidental findings rather than biologically aggressive disease.

Kaplan–Meier survival analysis showed no significant difference in survival between patients with and without nodules, and this finding persisted after adjustment for age, T stage, and N stage in a multivariable Cox proportional hazards model. In contrast, increasing age, advanced primary tumour stage, and nodal disease were independently associated with poorer survival. This is consistent with the established natural history of oral cavity squamous cell carcinoma, in which tumour burden and regional nodal spread remain the major determinants of prognosis [3]. The fact that pulmonary nodules were not independently prognostic after controlling for these factors supports the interpretation that many such nodules may represent incidental findings rather than a marker of occult systemic disease [11]. Expanded multivariable Cox proportional hazards analyses incorporating pathological risk factors demonstrated that perineural invasion and nodal disease remained independently associated with poorer overall survival, whereas lymphovascular invasion and margin status were not. Importantly, pulmonary nodules remained non-significant after adjustment for these established prognostic factors, further supporting the interpretation that most incidental pulmonary nodules identified during staging imaging may not represent biologically aggressive disease. Unfortunately, morphological descriptors such as ‘solid’ and ‘subsolid’ were often omitted in the current study’s radiology reports before 2016/17, thereby preventing morphological characterisation in the analysis.

The analysis of death from metastases produced a similar conclusion. Although the proportion of metastatic deaths was numerically higher among patients with nodules than among those without nodules, the difference was small and not statistically significant, and logistic regression did not demonstrate an independent association between pulmonary nodules and metastatic death. The calculated positive predictive value of nodules for metastatic death was modest (PPV = 30.3), which again argues against assuming that nodules detected on staging imaging are malignant by default. These findings suggest that small incidental pulmonary nodules alone should not necessarily alter curative treatment intent in otherwise appropriate patients.

The current study was not adequately powered to assess the longitudinal oncological significance of pulmonary nodules or the effectiveness of surveillance imaging strategies. Although a small number of patients who underwent appropriately timed follow-up imaging experienced favourable outcomes, only four patients met the predefined criteria for appropriate surveillance. Consequently, these findings should be regarded as exploratory and hypothesis-generating only. No robust conclusions can be drawn regarding the impact of follow-up imaging on survival or metastatic disease detection. Larger prospective studies incorporating standardised follow-up protocols are required to determine whether surveillance imaging influences clinical outcomes in patients with incidental pulmonary nodules identified during oral cavity SCC staging.

Contemporary management of pulmonary metastases has evolved considerably, with increasing consideration of surgical resection for selected patients, particularly those with p16-positive disease, and the emergence of immunotherapy as an additional treatment modality. These developments suggest earlier detection through interval imaging may offer clinical benefit. Larger prospective studies in modern treatment cohorts are therefore required to determine whether routine follow-up imaging may improve survival outcomes [15, 16].

Limitations of this study include incomplete or poorly filed documentation and the relatively small sample size. The study lost a significant number of participants due to part of their care being handled in other facilities, including staging thoracic imaging performed externally. Radiology reports were of variable quality. Radiographic interpretation was not based on standardised reporting characteristics, which may introduce heterogeneity; similarly, reporting was generated by a varied array of clinicians rather than uniform central review by a subspecialist thoracic radiologist. Morphological descriptors such as “solid”, “subsolid”, and other contemporary risk stratification features were frequently omitted, particularly in earlier reports, limiting detailed radiological characterisation of pulmonary nodules.

Several of the more clinically interesting subgroup analyses, particularly those relating to nodule size, multiplicity, and follow-up imaging, were based on small numbers and therefore lacked statistical power. The predominance of small pulmonary nodules within this cohort limits the generalisability of the findings to larger or radiologically suspicious lesions. Such lesions may have been classified as metastatic disease or synchronous primary lung malignancies during initial clinical assessment and therefore excluded from this study population.

Cause-of-death classification was performed retrospectively by a single investigator based on available clinical documentation and was not subject to independent adjudication, introducing the potential for outcome misclassification. Histological confirmation also represents a limitation of this study: only two pulmonary nodules underwent biopsy. Furthermore, distinguishing pulmonary metastases from oral cavity SCC and primary lung SCC may be challenging histologically, meaning that histologically confirmed squamous cell carcinoma within the lung parenchyma may have represented either metastatic oral cavity SCC or a synchronous primary lung malignancy.

Despite these limitations, the overall pattern of findings was clinically informative. In this selected cohort of patients with oral cavity SCC treated with curative intent, incidental small pulmonary nodules identified on staging imaging were not independently associated with metastatic death or reduced overall survival. However, given the predominance of small nodules and the limited number of oncologic outcome events, the study may have been underpowered to detect modest but clinically meaningful prognostic differences. Accordingly, the absence of a statistically significant association should not be interpreted as definitive evidence that pulmonary nodules lack prognostic significance in all clinical contexts.

These findings should not be extrapolated to larger or radiologically suspicious pulmonary lesions, which were uncommon within the present study. Furthermore, radiographic features of pulmonary nodules did not reliably identify a subgroup at markedly higher risk of death from metastatic disease. In contrast, the expected oncologic variables of age, tumour stage, nodal disease, and perineural invasion emerged as the principal determinants of survival.

Taken together, these findings suggest that clinicians should continue to prioritise established prognostic markers when counselling patients and planning treatment, rather than placing disproportionate emphasis on small incidental pulmonary nodules detected during staging investigations. Further prospective studies with standardised imaging and pathology reporting protocols, and larger cohorts are required to better define the prognostic significance of pulmonary nodules in head and neck cancer.

## Data Availability

All data supporting the findings of this study are available within the paper and its Supplementary Information.
